# Public Knowledge About COVID-19 Booster Vaccines in Pakistan: A Study Conducted in a Tertiary Care Hospital in Karachi

**DOI:** 10.7759/cureus.40284

**Published:** 2023-06-12

**Authors:** Misha Khan, Sidra Jabeen, Syed Khizer Ali, Muhammad Huzaifa Tofique, Muhammad Saad Shabbir, Rooha Baig, Muhammad Naheel Khalili, Satesh Kumar, Mahima Khatri, Giustino Varrassi, Fnu Sapna, Arjan Dass, Nomesh Kumar

**Affiliations:** 1 Medicine and Surgery, Liaquat National Hospital and Medical College, Karachi, PAK; 2 Neurosurgery, Liaquat National Hospital and Medical College, Karachi, PAK; 3 Medicine, Liaquat National Hospital and Medical College, Karachi, PAK; 4 Medicine and Surgery, Shaheed Mohtarma Benazir Bhutto Medical College, Karachi, PAK; 5 Medicine and Surgery, Dow University of Health Sciences, Karachi, PAK; 6 Pain Medicine, Paolo Procacci Foundation, Rome, ITA; 7 Internal Medicine, Detroit Medical Center, Detroit, USA; 8 Medicine and Surgery, Willis-Knighton Health System, Shreveport, USA; 9 Internal Medicine, Detroit Medical Center/Wayne State University Sinai-Grace, Detroit, USA

**Keywords:** hospital, booster, pakistan, vaccines, covid-19

## Abstract

The coronavirus disease 2019 (COVID-19) pandemic has deteriorated the healthcare system and economy worldwide. Globally, by making the primary vaccination against the coronavirus necessary, the surge in cases waned, but as the effects of this vaccination decreased after some time, to prevent another pandemic, vaccination was still necessary. As a result, receiving a COVID-19 booster shot can boost immunity against the coronavirus. This study aimed to assess knowledge of COVID-19 booster vaccines in Pakistan among the general public and understand the factors affecting the vaccination process in the state. In this cross-sectional study, non-probability convenience sampling was done. Its physical data collection was conducted in September 2022 in a tertiary care hospital in Karachi, Pakistan. Data were collected from 384 individuals who visited the hospital with consent before filling out the questionnaire. The mean age of respondents was 35.81 (standard deviation (SD) = ±13.006), and 98.7% of individuals were primarily vaccinated for COVID-19, but out of these, only 60.1% received the booster jab. The most commonly reported side effects of primary doses of COVID-19 and its booster were pain at the injection site, fatigue, and fever, but these effects did not appear to have as much of an impact on the vaccination process as education did. The results are evident that out of primarily vaccinated individuals against COVID-19, 40.16% are reluctant to receive its booster. Therefore, it is essential to create awareness among the masses about vaccination and its importance.

## Introduction

The discovery of new variants of coronavirus disease 2019 (COVID-19) created confusion among the general public all over the world. No one knows when the pandemic will end, but the new Omicron wave is said to be more infectious and less virulent [1]. Some health officials are hopeful that COVID-19 will become seasonal flu after this fifth wave [2], but vaccination, like other seasonal diseases [3], will still be required to gain immunity against coronavirus infections. In South Africa, on November 24, 2021, the first case of the Omicron virus emerged [4]. Just a few days later, the first case of the Omicron virus was confirmed in Karachi on December 13, 2021. Before this, the cases were declining, but in the following months, a sudden surge is observed in the number of cases [5]. New COVID-19 precautionary measures were implemented in the country [6]. The COVID-19 precautions are helpful and necessary, but immunity against COVID-19 can only be achieved through vaccinations. According to the National Command and Operation Center (NCOC) Pakistan, all citizens over the age of 18 are advised to take booster shots after six months of their COVID-19 vaccination course. The government conducted various awareness and healthcare programs during the COVID-19 outbreak.

In an observational study in Pakistan, refusal to get COVID-19 vaccination was seen in families with low income and people who lack formal education. Hence, sociodemographic status and education are significant factors affecting the vaccination drive [7]. To date, the goal of vaccinating the whole nation against COVID-19 has not been achieved. In addition, new variants are putting survival at greater risk. In a case-control study conducted in England, it was proven that in elderly people (over 50 years of age) who have completed their primary coronavirus vaccination course, an additional dose can increase immunization [8]. Similarly, in another study, all adults were recommended to get a booster dose to maximize protection against omicron and other variants [9].

The effectiveness of the COVID-19 vaccine declines after six months [10], so getting an additional dose can decrease the severity of the disease and the risk of death. After several trials and successful results, a booster vaccination drive started in the United Kingdom and several other Western states in September 2021. Later, amid rising Omicron cases, free booster vaccinations started in Pakistan on January 1, 2022. The government has been trying its best to get the situation under control since the start of the pandemic. Immunization against coronavirus improved the situation, and if the public cooperated with the government during this Omicron period, the virus’ spread could be halted.

An assessment of public knowledge about COVID-19 booster vaccines helped us understand the mass psychology and factors affecting the COVID-19 booster vaccination process in the state. Previous vaccination courses have helped in the eradication of diseases. Similarly, vaccination against the coronavirus and the administration of booster shots might help put an end to the pandemic.

## Materials and methods

Study design, sampling technique, and participants

In this cross-sectional survey, our sampling technique was a non-probability convenience sample. The study population consisted of the general population of Pakistan. All citizens of Pakistan above the age of 18 who had their first vaccination course for COVID-19 completed and visited a tertiary care hospital in Karachi were eligible to participate in the survey. Exclusion criteria included people who could not understand Urdu or the English language and foreigners. Participation was voluntary, and full consent was obtained from the participants. The anonymity of participants and confidentiality of personal data was ensured. Participants had the right to withdraw at any stage, and no harm or gain was caused to the participants by any means.

Sample size

The sample size was estimated using Raosoft software [11]. Our minimum sample size was 384. A confidence level of 95%, a 50% population proportion, and a 5% margin of error were used.

Data collection procedure

Data collection was done physically, through printed questionnaires. The form was available in both Urdu and English. Data were collected from patients and their attendants in all waiting areas and outpatient departments of a tertiary care hospital in Karachi. Around 400 people participated in the study, out of which 16 forms were excluded due to incomplete data. The final respondents were 384, with a response rate of 100%.

Instrument

We formulated a questionnaire consisting of sociodemographic questions, questions for the assessment of public knowledge about COVID-19 booster vaccines, and factors affecting the immunization program in the country.

Data analysis

The Statistical Package for the Social Sciences (SPSS) version 22 (IBM SPSS Statistics, Armonk, NY, USA) was used for descriptive data analysis and all calculations. The mean and standard deviation (SD) will be calculated for continuous variables such as age and family size. Frequency and percentage were calculated for categorical variables such as gender, residence, marital status, education, employment status, monthly income, comorbidity, coronavirus primary vaccination, and booster vaccination. A P value of <0.05 was considered significant.

## Results

Demographics

The overall response rate was 99.47%, and the majority of the participants were male (60.6%). The mean age of the respondents was 35.81 (SD = ±13.006). Most of them were married (63.8%), employed or self-employed (54%), undergraduates (26.6%) or graduates (37%), and dwellers of urban areas (90.6%) in Sindh province (84.6%), belonging to a stable socioeconomic class, i.e., 30.4% have a household income of PKR 75,000-100,000, and 46% of the population have PKR 100,000 or more. The demographic characteristics of the participants are represented in Table 1.

**Table 1:** Demographic characteristics of participants SD: standard deviation

Variables		Mean/%
Age		35.81 (SD = ±13.006)
Gender	Male	60.6%
	Female	39.4%
Province	Sindh	84.6%
	Punjab	8.6%
	Balochistan	5%
	Khyber Pakhtunkhwa	1.3%
	Azad Kashmir	0.3%
	Gilgit Baltistan	0.3%
Residence	Urban	90.6%
	Rural	9.4%
Marital status	Single	33.9%
	Married	63.8%
	Widowed	1.8%
	Divorced/separated	0.5%
Education	Cannot read or write	0.8%
	Can read only	0.5%
	Under matric	2.1%
	Matric	12%
	Intermediate	21.1%
	Undergraduate	26.6%
	Bachelors/masters/PhD	37%
Employment status	Employed/self-employed	54%
	Unemployed	22.2%
	Homemaker	18.8%
	Others	5%
Household income	Under Rs. 25,000	2.2%
	Between Rs. 25,000 and Rs. 50,000	8.6%
	Between Rs. 50,000 and Rs. 75,000	12.9%
	Between Rs. 75,000 and Rs. 100,000	30.4%
	Rs. 100,000 or over	46%

Side effects after getting COVID-19 vaccines and its effect on the public’s perception

From the total of 381 respondents, 379 received both doses of the COVID-19 vaccine. The reported side effects after receiving primary doses of the COVID-19 vaccine are summarized in Table 2.

**Table 2:** Side effects after receiving primary doses of the COVID-19 vaccine COVID-19: coronavirus disease 2019

Vaccination		Pain	Redness	Swelling	Body aches	Fatigue	Fever	Chills	Headache	Nausea
Sinopharm	1^st^ dose (157)	47 (29.93%)	10 (6.3%)	3 (1.91%)	13 (8.3%)	69 (43.94%)	28 (17.83%)	7 (4.45%)	8 (5.09%)	3 (1.9%)
	2^nd^ dose (156)	47 (30.12%)	7 (4.48%)	3 (1.92%)	9 (5.76%)	65 (41.66%)	25 (16.02%)	2 (1.28%)	9 (5.76%)	1 (0.64%)
Sinovac	1^st^ dose (113)	49 (43.36%)	4 (3.5%)	3 (2.65%)	15 (13.27%)	40 (35.39%)	29 (25.66%)	1 (0.88%)	9 (7.96%)	5 (4.42%)
	2^nd^ dose (113)	45 (39.82%)	3 (2.65%)	4 (3.53%)	14 (12.38%)	44 (38.93%)	21 (18.58%)	2 (1.76%)	10 (8.84%)	4 (3.53%)
Cansino	1^st^ dose (21)	5 (23.8%)	0 (0%)	0 (0%)	2 (9.5%)	5 (23.80%)	3 (14.28%)	0 (0%)	0 (0%)	0 (0%)
	2^nd^ dose (17)	3 (17.64%)	0 (0%)	0 (0%)	1 (5.88%)	6 (35.29%)	2 (11.76%)	0 (0%)	0 (0%)	0 (0%)
Sputnik	1^st^ dose (24)	6 (25%)	3 (14.28%)	6 (25%)	6 (25%)	12 (50%)	7 (29.16%)	2 (8.3%)	4 (16.66%)	4 (16.66%)
	2^nd^ dose (24)	6 (25%)	5 (20.83%)	5 (20.83%)	9 (37.5%)	11 (45.83%)	10 (41.66%)	4 (16.66%)	4 (16.66%)	2 (8.33%)
AstraZeneca	1^st^ dose (6)	3 (50%)	1 (16.6%)	1 (16.6%)	2 (33.3%)	3 (50%)	4 (66.6%)	0 (0%)	0 (0%)	0 (0%)
	2^nd^ dose (6)	2 (33.33%)	0 (0%)	0 (0%)	1 (16.66%)	3 (50%)	3 (50%)	0 (0%)	1 (16.66%)	1 (16.66%)
Pfizer	1^st^ dose (46)	16 (34.78%)	0 (0%)	0 (0%)	7 (15.21%)	12 (26.08%)	12 (26.08%)	1 (2.17%)	3 (6.5%)	1 (21.7%)
	2^nd^ dose (46)	17 (36.95%)	2 (4.34%)	1 (21.73%)	5 (10.86%)	5 (10.86%)	13 (28.26%)	1 (2.17%)	2 (4.34%)	1 (21.7%)
Moderna	1^st^ dose (14)	4 (28.57%)	0 (0%)	0 (0%)	6 (42.85%)	5 (35.71%)	6 (42.85%)	1 (7.14%)	4 (28.5%)	2 (14.28%)
	2^nd^ dose (14)	3 (21.42%)	0 (0%)	0 (0%)	4 (28.57%)	5 (35.71%)	6 (42.85%)	1 (7.14%)	4 (28.5%)	1 (7.14%)

It was found that out of 379 vaccinated individuals, only 228 (60.2%) received the booster dose. Post-vaccination side effects might be the reason for this noticeable difference in primary and booster dose recipients. These vaccination side effects are evident in Table 2. The collected data revealed that 94 (60.25%) of Sinopharm, 57 (50.9%) of Sinovac, nine (42.8%) of Cansino, 21 (87.5%) of Sputnik, five (83.3%) of AstraZeneca, 34 (73.9%) of Pfizer, and eight (57.14%) of those who received Moderna vaccines also received the booster dose for COVID-19. This indicates that more than the side effects, some additional reasons, such as public knowledge and perception, have negatively impacted the vaccination process.

Willingness to receive a booster dose of COVID-19

**Figure 1:**
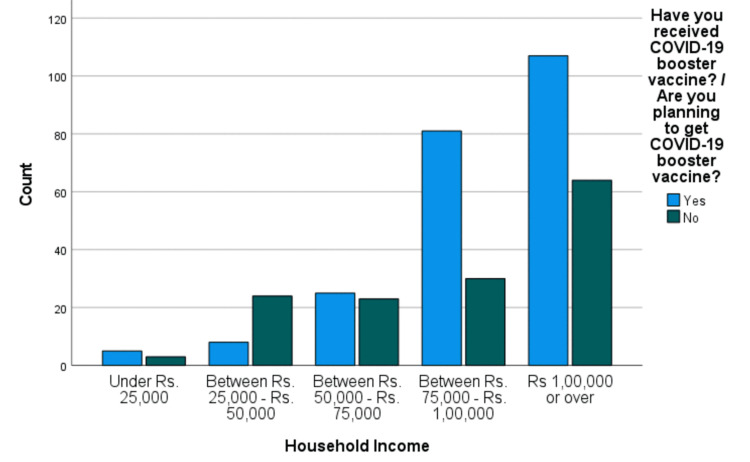
Graphical presentation of families with a household income of under Rs. 25,000, between Rs. 25,000 and Rs. 50,000, between Rs. 50,000 and Rs. 75,000, between Rs. 75,000 and Rs. 100,000, and Rs. 100,000 or over and their willingness to receive a booster vaccine COVID-19: coronavirus disease 2019

Our analysis revealed a significant relationship between people receiving the COVID-19 booster vaccine and recommending the COVID-19 booster vaccine (odds ratio (OR): 26.760, X2(1), 83.266, P < 0.0001). Similarly, employed or self-employed individuals were more likely than unemployed, homemakers, or others to receive the COVID-19 booster vaccine (X2(3), 8, P = 0.046). The primary vaccination for COVID-19 and its booster dose were made mandatory at most workplaces, which could be the reason why most booster-vaccinated people were employed. Furthermore, compared to low-income families, families with a household income of more than PKR 75,000 were significantly more likely to receive the COVID-19 booster vaccine (X2(4), 25.92, P < 0.0001), as shown in Figure 1.

**Figure 2:**
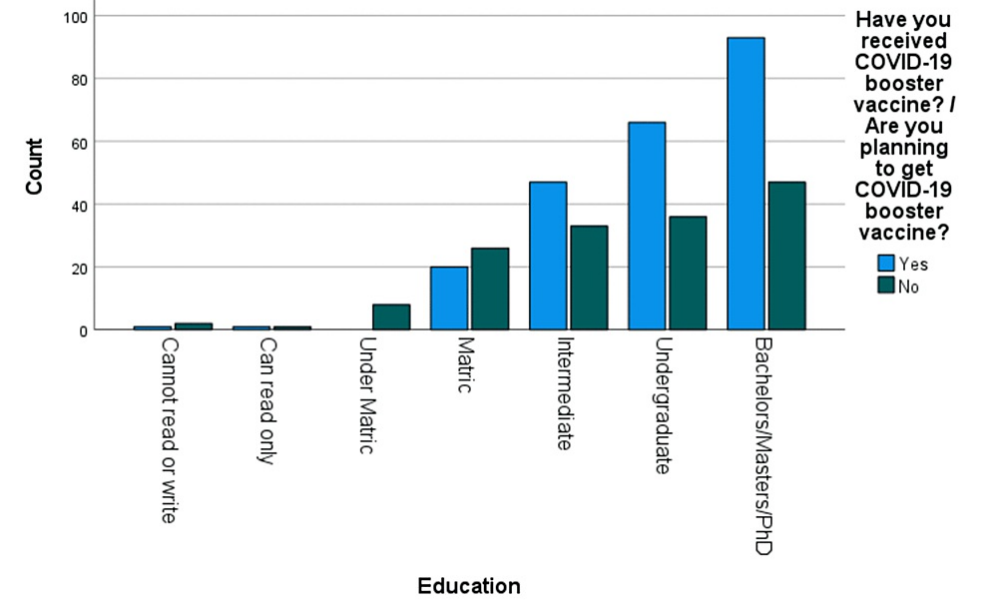
Graphical presentation of the relationship between the education level of people and their willingness to receive the COVID-19 booster vaccine COVID-19: coronavirus disease 2019

Also, a direct and significant relationship between the education level of people and their willingness to receive the COVID-19 booster vaccine (X2(6), 21.576, P = 0.001) was also determined, as shown in Figure 2.

Similarly, there is a significant relationship between people receiving the COVID-19 booster vaccine and those whose families have also received the vaccine (OR: 5.912, X2(1), 50.128, P < 0.0001). Also, 223 people out of 380 received the COVID-19 booster vaccine and also suggested people get the COVID-19 booster vaccine. However, 95 people did not receive the COVID-19 booster vaccine but advised others to get the booster dose (OR: 26.760, X2(1), 83.266, P < 0.0001).

Booster dose of COVID-19 and its side effects

Of the total of 228 people who received the COVID-19 booster vaccine, 63 experienced pain. There is a significant association between people receiving the COVID-19 booster vaccine and pain as a side effect (X2(1) = 5.59, P = 0.018). The relative risk is also 1.091, reflecting that the cohort that gets the booster shot is more likely to suffer from pain as a side effect. About 56 participants experienced fatigue after getting booster shots. There is a significant association between people receiving the COVID-19 booster vaccine and fatigue as a side effect (X2(1) = 4.788, P = 0.029). The relative risk is also 1.087, reflecting that the cohort that gets the booster shot is more likely to suffer from fatigue as a side effect. Fever occurred in 46 of the 228 people who received the COVID-19 booster vaccine. There is a significant association between people receiving the COVID-19 booster vaccine and fever as a side effect (X2(1) = 3.733, P = 0.053). The relative risk is also 1.082, reflecting that the cohort that gets the booster shot is more likely to suffer from fever as a side effect, as shown in Table 3.

**Table 3:** Side effects associated with the booster dose of COVID-19 vaccine COVID-19: coronavirus disease 2019

Symptom	N = 243	P value
Pain	63	0.018
Redness	18	0.258
Swelling	11	0.384
Body aches	31	0.125
Fatigue	56	0.029
Fever	46	0.053
Chills	3	0.655
Headache	23	0.196
Nausea	7	0.762

Factors affecting the COVID-19 booster process

**Figure 3:**
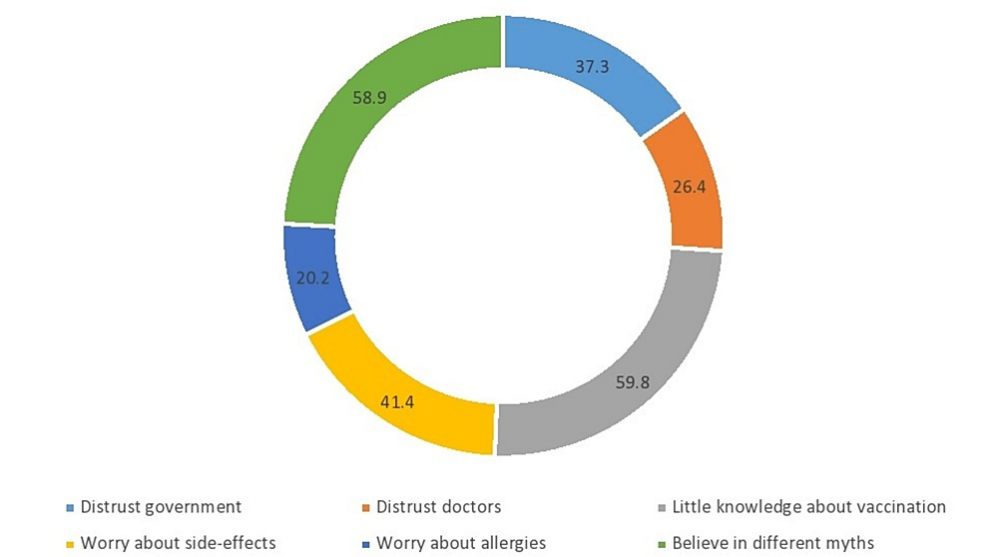
Visual representation of factors affecting the COVID-19 booster process COVID-19: coronavirus disease 2019

They believe that lack of vaccination knowledge is a factor affecting the vaccination process in Pakistan, as 153 of the 381 people received the COVID-19 booster vaccine. On the other hand, 76 people did not receive the booster vaccine because they had little knowledge about it (OR: 2.067, X2(1), 11.60, P = 0.001), shown in Figure 3. The infectivity for COVID-19 in the sample after receiving the booster was 13.4% (P = 0.336). Surprisingly, most of them were Pfizer recipients, which is one of the most effective doses. The death of a loved one due to coronavirus neither increased nor decreased the ratio of recipients of booster shots.

Coronavirus protocols

**Figure 4:**
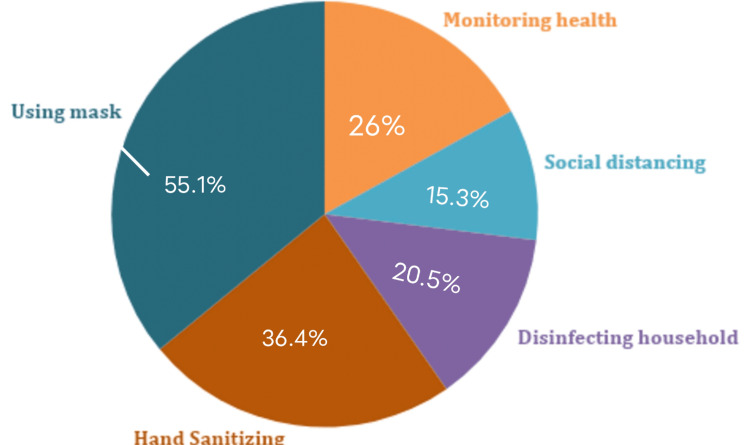
Graphical representation outlining COVID-19 safety measures COVID-19: coronavirus disease 2019

Various methods for COVID-19 safety measures are shown in Table 4 and Figure 4.

**Table 4:** COVID-19 safety measures COVID-19: coronavirus disease 2019

Variables	Frequency	Percentage
Hand sanitizing	140	36.4%
Using mask	212	55.1%
Covering cough and sneeze	116	30.1%
Cleaning and disinfecting household	79	20.5%
Avoiding crowded places	55	14.3%
Social distancing	59	15.3%
Monitoring health	100	26%

There has been a strong association between the education level of people and their care and concerns about hand sanitizing, with a direct relationship between the level of education and hand sanitizing (X2(6), 21.644, P < 0.001), the education level of people and them covering their sneezes (X2(6), 28.716, P < 0.00001), and the education level of people and their concern about social distancing (X2(12), 25.123, P < 0.014).

A strong association is also seen between people receiving a COVID-19 booster vaccine and sanitizing hands (OR: 4.181, X2(1), 34.844, P < 0.0001), using a mask (OR: 5.011, X2(1), 52.684, P < 0.0001), covering coughs and sneezes (OR: 7.498, X2(1), 50.356, P < 0.0001), sanitizing hands and recommending others get the COVID-19 booster vaccine (OR: 4.628, X2(1), 17.247, P < 0.0001), cleaning and disinfecting household items (OR: 8.980, X2(1), 37.428, P < 0.0001), observing social distancing (X2(2), 19.917, P < 0.0001), avoiding poorly ventilated and crowded places (OR: 10.229, X2(1), 27.339, P < 0001), and monitoring their health (OR: 11.756, X2(1), 55.626, P < 0.0001).

People who recommended getting COVID-19 booster vaccine were also highly seen covering coughs and sneezes (OR: 33.463, X2(1), 28.253, P < 0.0001), cleaning and disinfecting household items (OR: 4.375, X2(1), 8.907, P = 0.003), observing social distancing (X2(2), 8.535, P = 0.014), avoiding poorly ventilated and crowded places (OR: 5.866, X2(1), 7.331, P = 0.007), and monitoring health (OR: 13.157, X2(1), 19.984, P < 0.0001).

## Discussion

The results of our study reveal that the general population is still not as keen on taking the booster vaccine as one might think, with the percentage of people not taking the booster reaching 39.8%. This noncompliance of the general public despite multiple advertisements by the government can be credited to a number of reasons, such as perception and education.

People who are of high social value, i.e., people with formal education and a well-paying job, were more likely to take the booster as compared to those who are unemployed and housewives. Similarly, better compliance was seen in people with a household income of more than PKR 75,000 when compared to those belonging to lower-wage groups. This shines a light on the fact that education and access to financial freedom play a key role in determining people’s perceptions about taking the booster. This becomes more interesting as the booster was given free of charge, which means people from all wage groups had access to the same material. Despite this, some were willing and some were not.

A study conducted in the United Kingdom showed somewhat similar results, where they detected a pattern of unwillingness to take booster doses and lower education levels and employment rates [12]. However, the percentage of people who took the booster COVID-19 vaccine was 92.3%, which is higher when we compare it to the 59.84% that were in our study. This can be credited to the low levels of education and poor self-awareness among the public, with Pakistan having a literacy rate of 52% and the United Kingdom having a literacy rate of 90%.

In a study conducted in Peru to find out the percentage of the general population not getting COVID-19 boosters and their reasons, it was found that the people in rural areas had lesser compliance toward COVID-19 boosters as compared to those living in urban areas [13]. This was strange to see as data in Peru reported that COVID-19 in rural areas had more people dying compared to COVID-19 in urban areas. Still, people in rural areas were unlikely to get vaccinated. This can be attributed to the fact that education, employment rate, and healthcare accessibility played a major role in this. As the percentage of all these factors was considerably lower in rural areas, the percentage of people getting vaccinated was low too. Similarly, in our study, there is a direct link between people getting boosters and people who are employed and educated as it is likely that the institutions where these people are enrolled either to study or to earn a living had made COVID-19 vaccines and boosters mandatory.

Another study in Germany was conducted to check the reasons for noncompliance among the public [14]. The leading factor was the lack of information about the vaccine available to the public; many believed that the side effects were far greater than the benefits they would receive by taking the vaccine. In our study, a similar finding can be noted, as 76 people reported not taking the booster due to a lack of available information. This should be of importance, as it is the job of the government to make sure the public knows thoroughly what the vaccines are, what their side effects are going to be like, and the benefits of taking the vaccine.

Also, another factor to note down is that, in this study, the percentage of people that gave the reason for not receiving vaccines as they believed in natural immunity and also the fact that COVID-19 is just the seasonal flu was 22.9% and 26.4%, respectively. This can be compared to our study where 58.9% of people did not get the booster vaccine as they believed in myths. This pattern shows the alarmingly high rate of false knowledge and lack of education that is prevailing in society. Therefore, it is important for the government to do better advertisements for COVID-19 vaccines and make people aware of the facts.

A study in Pakistan checked the difference between the compliance of healthcare workers and the general public, and the results showed that even healthcare workers were unwilling to take the vaccine, although the percentage was very small (12.5%) [15]. More compliance, in general, was shown by healthcare workers in comparison to the general public, but a key thing to note here is that more than half the sample, i.e., 62.4%, chose imported vaccines over domestic products, with more healthcare workers choosing imported vaccines. This goes to show the lack of trust the people have in government, which can only be improved by spreading mass awareness. A similar trend was seen in this study as well; people with primary or no education at all were more likely to not take the vaccine as compared to their counterparts.

A Japanese study that aimed to check for compliance with vaccines among the general public showed some very interesting findings [16]. They asked the public whether they would even take the third booster if it would be available to the general public, and 78.3% of the people agreed, with 37.5% strongly agreeing to get the third dose. Comparing that to 39.8% of people who took boosters in our study, a visible difference can be seen, although the demographics of both studies were more or less the same. This might be because the difference in actions of both governments in the light of educating and spreading awareness to the public was prominent. However, a similar trend can be noted here that the percentage of people willing to choose the booster vaccine considerably dropped after they had developed certain side effects toward it, which showed that despite the efforts made by the authorities, an individual would not risk his/her life if he/she senses some danger from the vaccine.

A study in Greece to check for the predictors of compliance for vaccinations showed that the main reason for not getting booster vaccinations was the fact that the public was concerned about their health in the long run [17]. This can be seen in the percentage of people who showed a willingness to accept the vaccine, which was only 22.7%, with 39.3% of people being unsure but could be persuaded if presented with the right facts. A key factor to note here is that the demographics of the study were very different, with an average percentage of males being 23.9% compared with our study, which was more than 60%. Also, a main difference can be noted in the education level, with only 37% of the population having a master’s degree and 26.6% having an undergraduate university degree in our country, and 72.4% of people having a university degree in the sample in Greece.

Another study was conducted in Poland to find out whether there was any difference in booster compliance between healthcare professionals and medical university students [18]. The results showed that there was no major difference between the acceptance percentages in the first dose of the vaccines as both the sample population belonged to the same field. A similar trend could be seen for the boosters as well, and when asked for the reasons for acceptance, the majority answered with the safety of themselves and their family members. This goes to show the importance of the government’s role and education in the vaccine program.

We also bring to light the health disparities across the country and suggest implications for governments to target educational interventions that can reduce inequalities and improve health at a macro level. It was also seen that people with education and a sophisticated lifestyle actively sought out things that could ease their lives during the pandemic, things as basic as hand sanitization, wearing a mask, and social distancing, while those with no access to education gave no heed to these activities despite multiple studies showing the improvement of life due to these daily precautions. At the most basic level, education has a positive impact on personal health by increasing self-awareness and attempting to improve one’s own life.

The limitations of this study include its cross-sectional study design and a limited sample size due to the physical collection of data. For better reporting of figures, we did not consider electronic data collection, and we hope these results might help in understanding public psychology and the factors hindering the vaccination process.

## Conclusions

This study clearly shows that in order to combat diseases, it is integral to educate people about vaccination and its essence and assist them in overcoming their fears and distrust. The findings are appalling, as 40.16% of those who are primarily vaccinated for COVID-19 are hesitant to receive a booster. We believe that in order to prevent another pandemic, it is necessary to raise public awareness about the importance of vaccination. As a result, we urge the health sector and government officials to look into this matter and take steps for the betterment of the people.
